# South Indian Branch of the British Medical Association

**Published:** 1888-03

**Authors:** 


### SOUTH INDIAN BRANCH OF THE BRITISH NEDICAL ASSOCIATION.

*Severe Injury to the Spinal Cord.*—Brigade-Surgeon Sibthorpe related the case of a young man who was admitted into the General Hospital six days after a severe blow in the interscapular region, which was immediately followed by extensive paralysis. When admitted he was paralyzed in both upper and lower extremities; respiration was almost purely diaphragmatic, the accessory muscles of respiration also moving the upper part of the chest somewhat; there was anæsthesia below the nipple level, and in the upper extremities below the elbows. He gradually sank and died on the seventh day. A few hours before death the temperature began to rise rapidly, and finally reached 109° F. in the mouth. At the necropsy the anterior part of the body of the fourth cervical vertebra was found to be bruised, and the membranes of the cord were at this level injected; the cord appeared normal externally, but on section a patch of red softening, the size of a large pea, was seen in the centre, just below the third pair of cervical nerves. The cord appeared to be otherwise healthy. The internal organs were congested, especially the posterior border of the right lung.

